# Case of a Large Pedunculated Biliary Cholesterol Polyp With Osseous Metaplasia

**DOI:** 10.7759/cureus.12357

**Published:** 2020-12-29

**Authors:** Ibrahim Abukhiran, Judy Jasser, Ilham Farhat, Sarag Boukhar

**Affiliations:** 1 Pathology and Laboratory Medicine, University of Iowa Hospitals and Clinics, Iowa City, USA

**Keywords:** pedunculated, biliary, cholesterol, polyp, osseous metaplasia, gallbladder

## Abstract

Cholesterol polyps are the most common benign gallbladder polyps and are usually seen in a background of cholesterolosis. Rarely, they can harbor foci of osseous metaplasia, which is an event of uncertain clinical significance that might be confused with cholelithiasis clinically or radiologically. Herein we report the case of a 78-year-old female with a 1.8-cm pedunculated polyp arising in the gallbladder body. Histologic examination showed microscopic foci of osseous metaplasia, characterized by heterotropic bone trabeculae rimmed by osteoblasts and surrounded by osteoclast giant cells. To the best of our knowledge, this case is the third case report of a cholesterol polyp with osseous metaplasia in the English literature. We also review the relative pathogenesis, clinical and pathologic findings, and previous reports.

## Introduction

Cholesterolosis is the accumulation of neutral lipid within subepithelial macrophages of lamina propria of gallbladder. Cholesterolosis is a common finding seen in 16% of cholecystectomies, frequently with cholesterol gallstones [1-3]. The pathophysiology of cholesterolosis is poorly understood; however, it is thought to reflect increased liver synthesis of lipids or bile supersaturation with cholesterol together, allied with enhanced absorption and esterification by the subepithelial macrophages and gallbladder epithelium. Polypoid cholesterolosis or cholesterol polyps are morphologic variations of the same diffuse process of cholesterolosis that occur when the lipid-filled histiocytic deposits grow larger and protrude into the lumen, forming a polyp [4]. Herein we report a case of a large pedunculated cholesterol polyp with microscopic foci of osseous metaplasia.

## Case presentation

**Figure 1:**
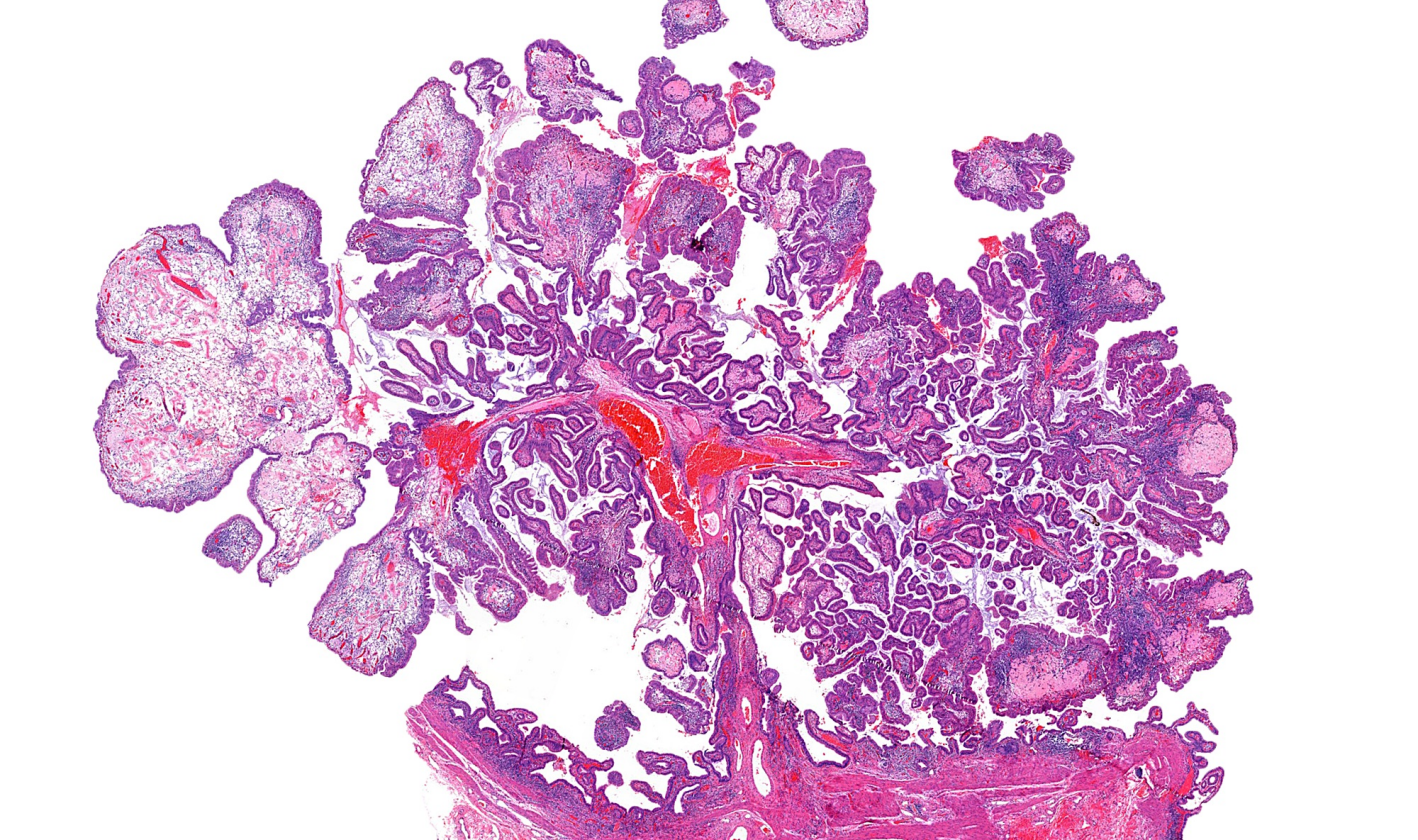
Gallbladder cholesterol polyp with heterotropic bone formation A low-power view showing a large pedunculated polyp with a lobulated architecture and variable number of branching villous projections indicated by the black arrow (H&E, ×2 magnification). H&E, hematoxylin and eosin

**Figure 2:**
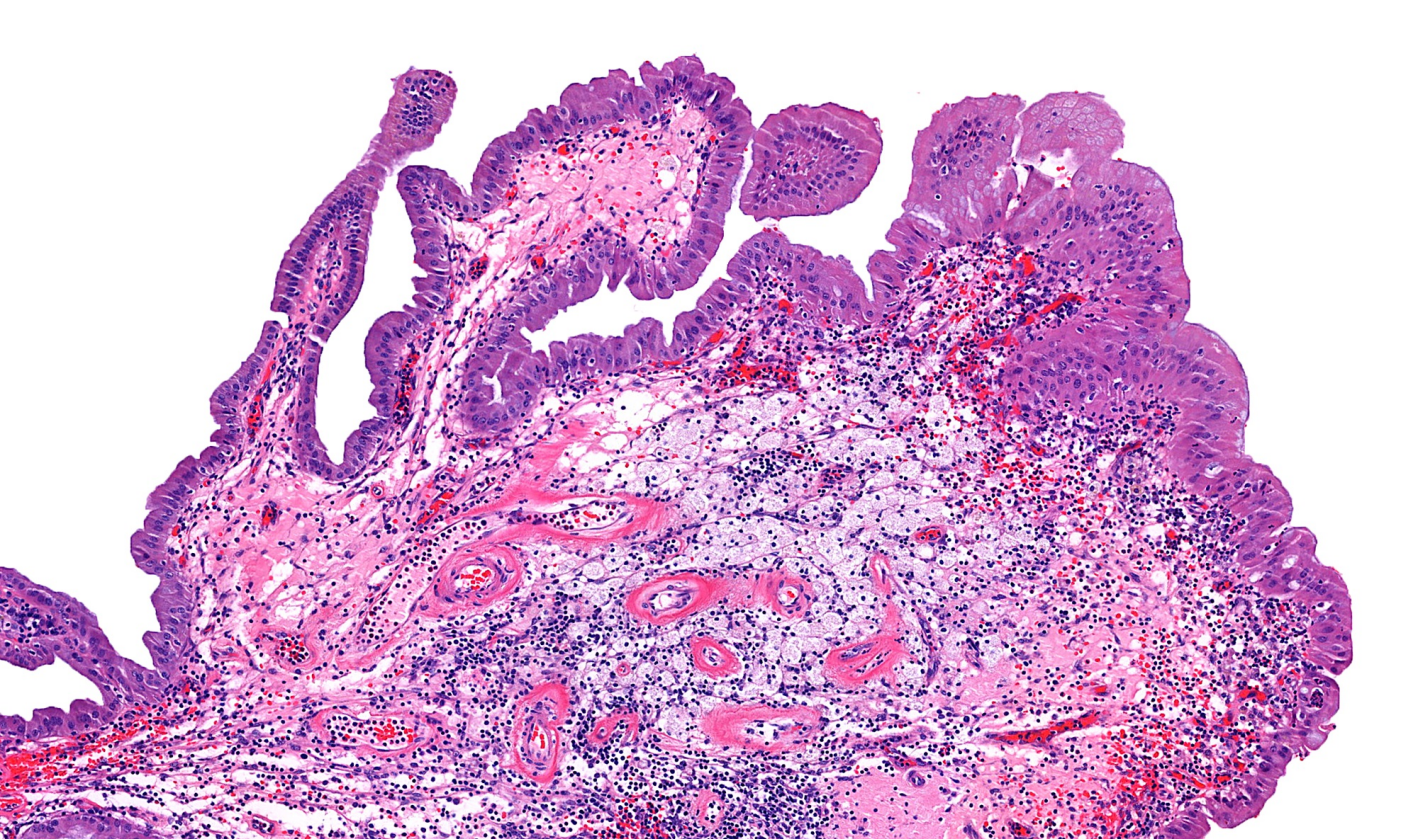
Gallbladder cholesterol polyp with heterotropic bone formation (lobulated architecture) Higher power view shows these lobules to be lined by histologically unremarkable biliary epithelium, with numerous foamy macrophages (black arrow) occupying the lamina propria, identical to that morphology seen in diffuse cholesterolosis (H&E, ×10 magnification). H&E, hematoxylin and eosin

**Figure 3:**
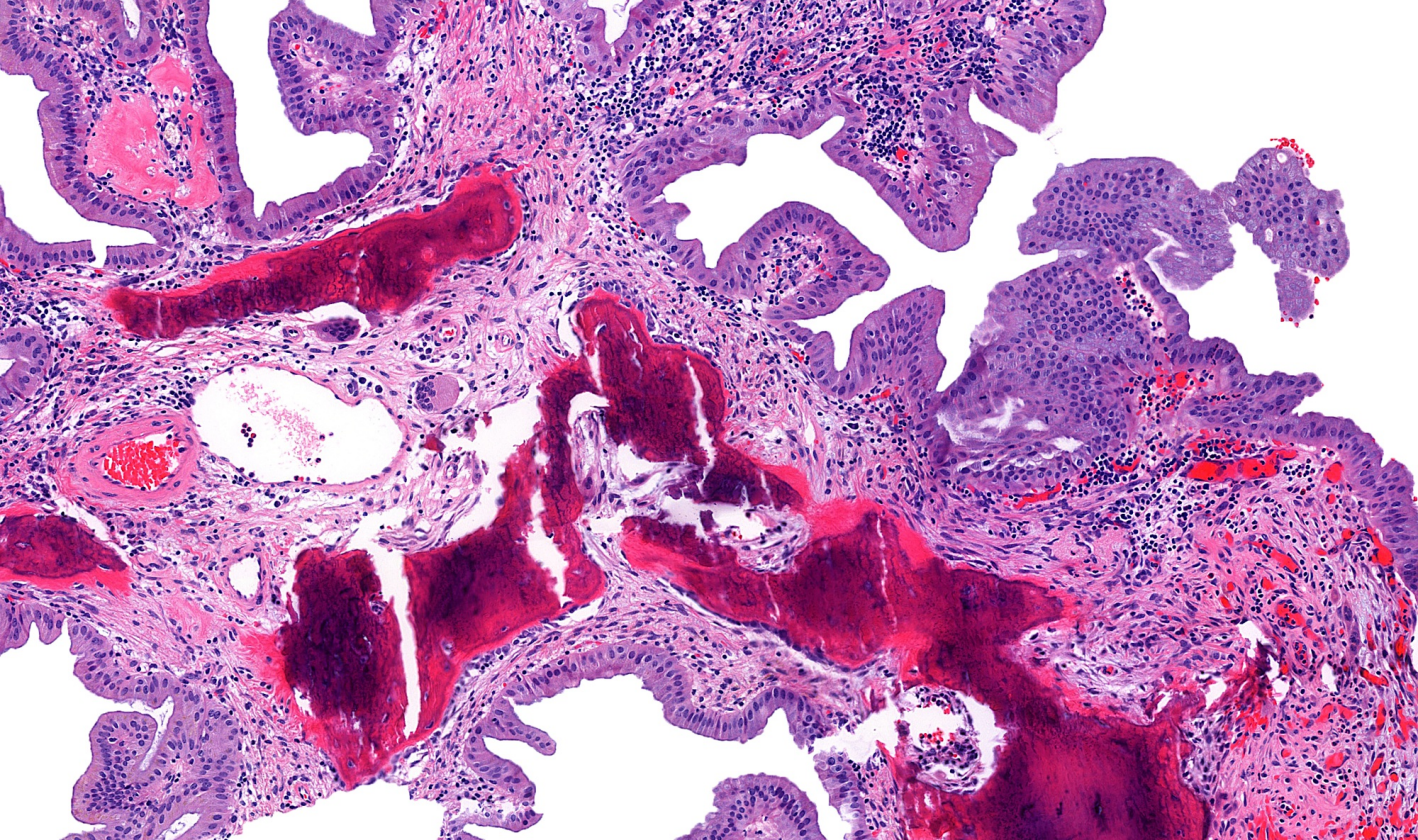
Gallbladder cholesterol polyp with heterotropic bone formation (osseous metaplasia) Other area of the polyp showing benign osteoid formation rimmed by osteoblasts and surrounded by osteoclastic multinucleated giant cells indicated by the black arrow (H&E, ×10 magnification). H&E, hematoxylin and eosin

A 78-year-old female presented to our institution for the evaluation of a gallbladder polyp and pancreatic intraductal papillary mucosal neoplasm (IPMN), which were radiologically identified during a workup for bilateral ovarian masses. She underwent cholecystectomy and bilateral oophorectomy that revealed bilateral serous cystadenofibromata. Gallbladder examination revealed a non-thickened wall with yellow-orange mucosa and a 1.8-cm pedunculated polyp arising in the gallbladder body. The lumen contained viscous, dark green bile with numerous black pigment stones. Histologic examination (Figures 1-3) showed a large lobulated polyp with branching villous projections that were lined by simple nondysplastic biliary epithelium and filled with foamy macrophages. Microscopic foci of osseous metaplasia are identified within polyp, characterized by heterotropic bone trabeculae rimmed by osteoblasts and surrounded by osteoclast giant cells. The patient did not receive additional treatment after the cholecystectomy. Her IPMN did not show high-risk characteristics for malignant transformation (such as presence of a mural nodule or associated pancreatic duct dilatation), and therefore, no treatment was given.

## Discussion

Gallbladder polyps have an estimated prevalence of 5% in the global population [5,6]. Of these, cholesterol polyps are the most common benign gallbladder polyps, accounting for 50%-60% of all gallbladder polyps. They are more prevalent in patients with cholesterolosis, morbid obesity, and in adult females between the fifth and sixth decades of life [1-3,7]. Cholesterol polyps usually form by an asymptomatic process and are typically discovered incidentally either on imaging or at the time of histological examination after cholecystectomy for unrelated reasons [8,9]. Rarely, patients with cholesterol polyps present with obstructive jaundice due to blockage of the distal bile ducts by detached polyp fragments [9,10].

Grossly, cholesterolosis appear as yellow streaks on the gallbladder mucosa, the color of lipid droplets within lamina propria, reminiscent of the surface of a strawberry, and called “strawberry gallbladder” [11]. When polyps form, they can grow as sessile polypoid excrescences on the mucosal surface, or they can be pedunculated and connected to the mucosa by a stalk, most of which are limited to 1.0 cm [12,13]. Interestingly, the bile itself can be thick-tarry with detached yellow flecks consisting of collections of foamy macrophages (lipoidic corpuscles).

Histologically, cholesterolosis refers to accumulation of foamy macrophages that fill the lamina propria. The lesional macrophages have small, dark nuclei with foamy (lipid-filled) cytoplasm. These changes are restricted to the gallbladder, without an extension to extrahepatic bile ducts. Cholesterol polyps have a lobulated architecture with a variable number of branching villous projections. These lobules are filled with foamy macrophages identical to the ones seen in diffuse cholesterolosis. The lining biliary epithelium is histologically unremarkable, and if pedunculated, the stalk is composed of a vascular connective tissue. The presence of lipofuscin pigment within histiocytes or the adjacent gallbladder epithelium is not uncommon. The gall bladder wall shows minimal inflammation unless associated with cholelithiasis. Osseous metaplasia (heterotropic bone formation) within cholesterol polyps has rarely been reported in the English literature (Table 1) [14-16]. Bone metaplasia of the gallbladder is a rare event of unknown clinical significance, though it might affect treatment options if confused with cholelithiasis clinically or radiologically.

**Table 1:** Reported cases of cholesterol polyps with osseous metaplasia

Author, year	Ortiz-Hidalgo & Baquera-Heredia, 2000 [14]	Ahn et al., 2016 [15]	Our case, 2020
Patient demographics	33-year-old female	26-year-old male	78-year-old female
Presenting symptoms	Abdominal pain	Abdominal pain	Incidental
Anatomic location	Body	Body	Body
Size (size of polyp or bone metaplasia)	0.3 cm	0.6 cm	1.8 cm
Polyp architecture	Pedunculated	Sessile	Pedunculated
Type of formed bone	Mature lamellar bone	Mature lamellar bone	Mature lamellar bone
Osteoblasts rimming	Present	Present	Present
Osteoclast-like giant cells	Absent	Absent	Present
Hematopoietic marrow cells	Absent	Absent	Absent
Background gallbladder mucosa	Polypoid cholesterosis, chronic cholecystitis	Diffuse wall thickening, chronic cholecystitis	Cholesterosis, chronic cholecystitis
Cholelithiasis	Present, mixed gallstones	Present, black pigment stones	Present, black pigment stones

In the gallbladder, osseous metaplasia has been first described to occur with adenocarcinoma [17]. It has also been reported to occur in benign gallbladder and mostly associated with chronic cholecystitis and cholelithiasis [16,18]. Due to the rarity of this condition, the exact incidence is not known. It has been theorized that it arises due to repetitive mucosal injury predisposing the gallbladder to dystrophic calcification that subsequently creates a favorable microenvironment for bone formation. In extra-skeletal tissues, the phenomenon of osteogenesis starts with undifferentiated mesenchymal cells that differentiate into osteoblasts. Osteoblasts then form the uncalcified organic matrix that will subsequently be filled with mineral salts [18].

The differential diagnosis of cholesterol polyps includes hyperplastic or inflammatory polyps. However, these two don’t have the lipid-filled macrophages and can have inflammation instead. Most gallbladder polyps are benign and require no surgical excision [12,13]. Cholesterol polyps, hyperplastic polyps, and inflammatory polyps are all benign and require no further treatment. Only 5% of gallbladder polyps (adenoma and adenocarcinoma) require surgical removal via cholecystectomy [13]. However, as gallbladder adenocarcinoma can be found incidentally in cholecystectomy specimens (in less than 1%), it has also been reported to arise and be confined to cholesterol polyps [19,20].

## Conclusions

Cholesterol polyps are the most common benign gallbladder polyps. Usually they are clinically asymptomatic and found incidentally on imaging or cholecystectomy specimens in a background of cholesterolosis. Rarely, they can harbor foci of osseous metaplasia, which is a rare event of unknown clinical significance, though it might affect treatment options if confused with cholelithiasis clinically or radiologically. We reported a case of a large pedunculated cholesterol polyp with microscopic foci of osseous metaplasia, characterized by heterotropic bone trabeculae rimmed by osteoblasts and surrounded by osteoclast giant cells. Herein, we also reviewed the pathogenesis, clinical and pathologic findings, and previous reports.
